# Structuring the World: Statistical Learning From the Top and From the Bottom

**DOI:** 10.1111/ejn.70712

**Published:** 2026-10-21

**Authors:** Alejandro Tabas, József Fiser, Máté Lengyel, Livia de Hoz

**Affiliations:** ^1^ Basque Center on Cognition, Brain and Language Donostia‐San Sebastian Spain; ^2^ Max Planck Institute for Human Cognitive and Brain Sciences Leipzig Germany; ^3^ Ikerbasque, Basque Foundation for Science Bilbao Spain; ^4^ Department of Cognitive Science Central European University Vienna Austria; ^5^ Department of Engineering University of Cambridge Cambridge UK; ^6^ Department of Physiology, Development and Neuroscience University of Cambridge Cambridge UK; ^7^ Neuroscience Research Center Charité Universitätsmedizin Berlin Berlin Germany; ^8^ Bernstein Center for Computational Neuroscience Berlin Germany

**Keywords:** Bayesian brain, context inference, learning in development, sensory processing, statistical learning, subcortical auditory processing

## Abstract

Statistical learning is the brain's primary strategy for transforming continuous, noisy, and ambiguous sensory inputs into structured internal representations. This process is studied through a diverse array of perspectives spanning different species, sensory systems, levels of analysis, and methodological frameworks. Here, we present four such perspectives. Two of them approach the problem from the bottom, examining the neural substrates of the biological implementation. The first line describes implicit contextual learning in the rodent auditory midbrain, showing how neuronal frequency tuning adapts to predictable statistical regularities over days. The second line shows that explicit knowledge modulates belief updating as early as the human auditory midbrain and thalamus, revealing a functionally specialized subcortical inferential hierarchy. The other two research lines approach it from the top, examining the computations the brain performs. The third line compares statistical computations across species and human development, uncovering fundamental differences in probabilistic chunking strategies between honeybees, human infants, and adults. The fourth line provides a computational framework for continual learning in which experience is organized into structured internal models through ongoing contextual inference. We then examine what each line can contribute to the others. We argue that, if the brain evolved statistical learning under pressure to behave optimally, this optimality must have been achieved globally through the constraints and synergies that emerge from the interplay between subsystems and levels of analysis. The neuronal mechanisms implementing a computation are shaped by the computation they perform, while that computation is itself constrained by the available physiology: a mutual dependence that can only be understood by bridging across perspectives.

## Graphical abstract

Statistical learning is the brain's primary strategy for transforming continuous, noisy sensory input into structured internal representations. We review four research perspectives on this process and conclude that understanding it fully requires integrating bottom‐up neural substrates with top‐down computational principles.

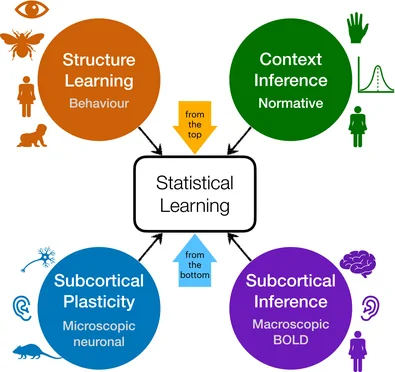

**Abbreviations**
BOLD: blood‐oxygen‐level‐dependent
COIN: contextual inference model of motor learning
fMRI: functional magnetic resonance imaging
IC: inferior colliculus
MGB: medial geniculate body
SL: statistical learning

## 1 Introduction

Humans and other animals access the external world through their senses. Although sensory inputs are continuous, noisy, and generally ambiguous, internal representations of the world are structured, discrete, and generally unambiguous (Radulescu et al. 2021). The brain exploits statistical regularities in the environment to structure this mapping (Fiser 2009), and these regularities must themselves be learnt from the continuous stream of sensory experience.

Human behavioral learning has traditionally been investigated within three largely separate research traditions. The first, *perceptual learning*, focuses on relatively simple forms of supervised training that lead to improvements of fine perceptual discrimination (Karni and Sagi 1991; Goldstone 1998; Fahle 2005). The second, *statistical learning* (SL), was developed to study implicit structure learning (Saffran et al. 1996; Newport and Aslin 2004; Conway and Christiansen 2005). A third, broader tradition, often referred to as *abstract learning*, encompasses forms of learning involved in acquiring knowledge about complex structures such as objects, categories, concepts, and grammars (Reber 1967; Smith and Medin 1981; Knowlton et al. 1994). More recently, however, SL has emerged as a powerful experimental and computational framework for investigating the implicit aspects of all three traditions. Here, we use the term SL in this broader sense, referring to the ability to implicitly detect and learn the statistical regularities of the environment across perceptual, statistical, and abstract domains (Fiser and Lengyel 2022).

Statistical learning is studied across a remarkable breadth of perspectives: different species (insects (Avargues‐Weber et al. 2020), rodents (McAlpine and de Hoz 2023), primates (Rey et al. 2022), humans (Heald et al. 2021), birds (Lu and Vicario 2014; Chen and Ten Cate 2015; Santolin et al. 2016, 2020; Fehér et al. 2017), etc.); different sensory modalities (visual (Fiser and Aslin 2001), auditory (Chen et al. 2023), somatosensory (Heald et al. 2021), olfactory (Davis 2004)); and different levels of analysis (computational modelling (Lengyel et al. 2019), behavioral analyses (Lengyel et al. 2021), neuroimaging (Tabas and Von Kriegstein 2024), and neural recordings (Cruces‐Solís et al. 2018)). Research further spans neurotypical processing (Tabas et al. 2020), developmental trajectories (Janacsek et al. 2012), and clinical populations (Roser et al. 2015).

Here, we present four perspectives currently used to study statistical learning in the brain. Each of the four perspectives reflects the line of work of each of the four authors; together, they aim to describe a nonexhaustive but representative set of lines currently used to study statistical learning.

Two examine the problem from the bottom, focusing on neural substrates, low‐level computations, and the role of early sensory areas. They constrain our understanding of the implementation of statistical learning in the brain by looking into the phenomenology of its neural implementation, but do not ask about the computational operations that result from this process.

The other two lines enquire into statistical learning from the top, identifying its underlying computations from a normative standpoint. They describe what the brain can learn and the specific mathematical operations that underlie and result from this learning, without enquiring about the neural implementation of such operations.

After a brief introduction to each of the four perspectives, we reflect on possible avenues in which these individual lines of research may complement and inform each other. We reason that the neural substrate of statistical learning constrains the computations performed by the brain, and that the necessities of the latter condition the former. We conclude that statistical learning can only be fully understood by integrating from‐the‐bottom and from‐the‐top perspectives.

## 2 Implicit Contextual Learning in the Auditory Midbrain

Statistical learning is a fundamental process for survival. The traditional view often localizes SL to high‐level associative areas like the cortex and hippocampus. However, accumulating evidence from the auditory system strongly suggests that SL might emerge early, within subcortical structures, where it potentially plays a critical role in optimizing the incoming sensory stream before it reaches the cortex (Cruces‐Solís et al. 2018; Chen et al. 2023; Robinson et al. 2016). This early adaptation suggests that adaptive coding and an emerging SL are unified manifestations of the nervous system's continuous sensitivity to environmental statistics (Maravall and De Hoz 2025).

This sensitivity operates across multiple timescales, ranging from seconds (short‐term adaptation) to days (long‐term consolidation) to the lifespan. Early adaptation of this kind sets processing cadences for learnt listening, enabling rapid sensory gating and filtering before detailed cortical processing begins.

A prime example of this early SL was found in the mouse inferior colliculus (IC), the main sensory convergence site in the auditory midbrain that receives extensive ascending sensory input and descending multimodal feedback. We found a remarkable sensitivity by IC to unsupervised sound‐context associations learnt over days (Cruces‐Solís et al. 2018). Animals lived in an enriched environment where they could enter one room activating a predictable sound every time. Plasticity was induced by this sparse stimulation in the form of discontinuous exposure to sound‐context associations presented minutes to hours apart. Plasticity developed slowly, emerging over 6 days of exposure, and was accompanied by long‐term changes involving gene and protein expression (Cruces‐Solís et al. 2018). It was evident in a shift in frequency tuning that spanned a large portion of the IC's tonotopic map and translated into an increase in neuronal frequency discrimination derived from the IC's frequency population vectors (Chen et al. 2023). There are three aspects in these data that suggest that the sensitivity to context‐sound associations in IC is an emerging form of SL. The first is that this plasticity depended on the predictability of the context‐sound associations and was not observed in animals exposed to no salient sounds or unpredictable associations. The second is that it was unsupervised and was independent of the presence of reward in the room. The third is a potentially time‐dependent role of top‐down feedback, as the effect of corticofugal inactivation early on during learning (days 1–6), when IC plasticity was only weakly expressed, was more pronounced than later on (Chen et al. 2023).

Hearing is an immersive experience that requires continuous separation between relevant and more or less noisy signals from dynamic backgrounds (de Hoz and McAlpine 2024). The IC might be well positioned to learn and represent predictable contextual sounds when the animal is immersed in a relevant acoustic environment and thus help segregate background from foreground. It is very likely though that this learning to segregate results from interactions within a cortico‐subcortical loop (McAlpine and de Hoz 2023).

## 3 Explicitly Learned Priors Drive Perceptual Processing in the Human Auditory Midbrain and Thalamus

Statistical learning is traditionally concerned with implicit processes (Fiser and Aslin 2001; Fiser 2009) where generative models are slowly extracted from environmental regularities. Once learned, these regularities can be deployed to form priors that inform perceptual inference (Friston 2003; Keller and Mrsic‐Flogel 2018; Walsh et al. 2020). In the auditory system, implicit priors shape these inferential processes at stages as early as the subcortical levels; see (Malmierca et al. 2015; Carbajal and Malmierca 2018; Tabas and von Kriegstein 2021) for reviews.

However, humans often rely on explicitly acquired knowledge, which can generate structured priors with far greater flexibility and speed than implicit learning alone (Bévalot and Meyniel 2024). This line of work examines whether explicitly acquired priors, typically attributed to cortical systems, can penetrate the sensory hierarchy and modulate inference at its earliest, subcortical stages, and how these interact with the implicit priors acquired through statistical learning.

To test this, we adapted the classical oddball paradigm so that explicit rules imposed position‐specific priors on deviant occurrence (Tabas et al. 2020). Participants were informed that each sequence contained a single deviant restricted to positions 4, 5, or 6, and were asked to report its position. This information induces deterministic evolution of priors across the sequence. For example, after three standards, the deviant is in position 4 with probability 1/3; after five standards, the deviant is in position 6 with probability 1. This paradigm dissociates belief update signals derived from implicit repetition statistics from those imposed by explicit, instruction‐based priors.

We used high‐resolution fMRI to quantify macroscopic responses to the deviant at each possible position, expecting responses to scale negatively with the predicted probability of the occurring stimulus. Larger belief updates should produce stronger BOLD signals. Results confirmed this pattern: BOLD responses in human auditory midbrain and thalamus varied inversely with the explicit prior. Least‐expected deviants (position 4) elicited the largest responses, whereas fully expected deviants (position 6) produced responses indistinguishable from repeated standards. These results demonstrate that explicit priors shape belief updating in the human auditory system already at very early processing stages. The effect replicates across pure tones, harmonic complex tones, and frequency‐modulated sweeps (Tabas et al. 2020, 2024; Tabas and Von Kriegstein 2024; Ara et al. 2024), indicating that explicit priors influence subcortical inference not by altering tonotopic drive but by modulating the generative model that thalamus and midbrain use to process sensory input.

However, explicit priors do not completely overwrite implicit priors learnt through statistical learning. The same stimulus sequences also instantiate a local implicit prior derived from repetition statistics: deviants occur infrequently relative to standards, and this imbalance provides a stable expectation that a standard is the most likely upcoming event. Subcortical BOLD responses reflect belief updating with respect to both prior sets: Explicit abstract priors and implicit local priors contribute additively to the computation. Explicit knowledge therefore enhances but does not replace the representational structure built from sensory statistics (Tabas and Von Kriegstein 2024; Tabas and von Kriegstein 2021).

This approach can also explore belief updating beyond feature‐based expectations. We have recently shown that explicit priors on stimulus presence are updated as early as the auditory thalamus, but not in auditory midbrain (Tabas et al. 2025). This reveals a division of labor along the subcortical pathway: whereas stimulus features are inferred in midbrain, stimulus presence is not inferred until thalamus.

Together, the findings from this research line position the subcortical auditory pathway as a distributed inferential system in which explicit and implicit priors jointly shape early sensory representations through multiple, functionally specialized computations.

## 4 Structuring Learned Representations Across Complexity, Development and Species

Originally, the term “statistical learning” was coined in the context of auditory word segmentation in infants (Saffran et al. 1996); investigations of this type of learning quickly expanded to other modalities including vision (Fiser and Aslin 2001; Kirkham et al. 2002; Zhao et al. 2013; Lee et al. 2021), where the focus shifted from explaining specific segmentation tasks to investigating SL as a general mechanism of forming representations (Fiser and Lengyel 2022). Early visual SL studies focused on spatial patterns composed of simple elementary shapes that formed an “inventory” of fixed combinations of two, three, or four shapes arranged in stable spatial relationships (chunks). These chunks were then randomly composed into complex multishape scenes in which chunk boundaries were not readily segmentable. Studies exposing naïve observers to a large number of such scenes established that human adults and 8‐month‐old infants automatically and implicitly extract the underlying chunks, whether chunk structure is defined by (a) higher co‐occurrence (joint probability of shape appearances), (b) higher predictability between chunk elements (conditional probability of shape appearances), or (c) higher independence of chunks (i.e., nonembeddedness of smaller chunks within larger chunks) (Fiser and Aslin 2001, 2002, 2005). Computational analyses demonstrated that although different computational mechanisms could, in principle, account for subsets of these results, only probabilistic chunking models were able to fully capture human behavior once embeddedness effects were taken into account, outperforming both counting models and probabilistic pairwise associative learning schemes (Orbán et al. 2008).

Recent developments in the field move beyond the level of embeddedness to explore the potential of SL as a framework for describing learning in the brain. Studies extending SL into the domain of structure learning have shown that abstraction does not stop at embeddedness. For example, simple implicit learning through exposure to scenes composed of chunk‐based inventories in which all chunks share a horizontal arrangement of elements does not merely result in implicit knowledge of the specific chunks themselves. When, after the exposure, the shape tokens in the task are replaced with an entirely new set of shapes, stronger—but still implicit—learners immediately transfer the abstract notion of “horizontalness,” showing enhanced learning of new chunks with horizontal structure relative to other structures (transfer learning). In contrast, weaker learners initially show the opposite pattern, exhibiting an interference effect by learning alternative structures more readily. Notably, following sleep‐dependent consolidation, these weak learners automatically restructure their acquired implicit knowledge and come to exhibit the same learning patterns as the strong learners (Garber and Fiser 2025). Other studies have demonstrated zero‐shot learning, in which purely visual SL exposure transfers immediately to sensorimotor control representations, and vice versa (Lengyel et al. 2019). Still others have shown that purely observational SL engages attentional mechanisms similar to those underlying object‐based attention (Lengyel et al. 2021). Together, these findings indicate that the outcomes of automatic SL generalize to structure learning and transfer and that SL is sufficient to support the formation of multimodal, object‐like representations through abstraction.

The emergence of such complex knowledge during SL has prompted the re‐evaluation of the computational interpretations of behavioral learning results. Historically, computational approaches to the perceptual, statistical and abstract learning traditions were treated as largely distinct, differing in their goals, methods, result types, and proposed underlying mechanisms. Recent analyses, however, suggest that this separation reflects methodological choices and task‐specific goals of the different fields rather than fundamental mechanistic differences. Under natural conditions, these forms of learning operate jointly, with efficient sensory coding and structure discovery occurring simultaneously at multiple representational levels. Consequently, experimental paradigms and computational models must be capable of capturing situations in which efficient encoding of sensory input and the discovery of structure, both at the level of latent descriptors directly linked to the input and at higher representational levels, operate together to support optimal learning (Fiser and Lengyel 2019, 2022).

In parallel with work emphasizing the integration of mechanisms underlying the three types of learning, an opposing perspective has emerged in the computational modelling literature, grounded in both computational and empirical studies. These studies emphasize that learning is not monolithic: The same learning performance can be achieved by very different computational learning algorithms, implemented through and supported by distinct forms of underlying knowledge stored in the brain (Bitterman 1975; Smith et al. 2012). Consequently, new methods are required that can specifically identify both the learning algorithms employed and the knowledge acquired during the learning process.

Investigations across humans and honeybees support this conjecture. For example, one spatial visual statistical learning study systematically compared implicit learning of sensory structure defined by element frequencies, joint probabilities, and conditional probabilities of spatially specific co‐occurrence between shape pairs in multielement displays, using the same experimental paradigm and stimulus sets across honeybees, human adults, and infants (Avargues‐Weber et al. 2020). Here, a 100% joint probability means that “whenever both A and B appear in a scene, Shape A always appears to the left of Shape B,” whereas a 100% conditional probability means that “whenever Shape A appears, Shape B always appears to its left.” While honeybees were able to learn implicitly and generalize from experience to novel situations as successfully as humans, their learning followed a markedly different pattern. Specifically, bees progressed by first learning elementary statistics and then pairwise statistics, but never showed evidence of learning conditional probabilities. In contrast, human adults and infants consistently learnt the most reliable chunks based on prior experience, becoming sensitive to larger chunks first and only later, if at all, to embedded substructures (Avargues‐Weber et al. 2020). In particular, adults maintained sensitivity to elementary statistics (i.e., differences in individual element frequencies), whereas infants did not (Fiser and Aslin 2001, 2002). These results can be reconciled by assuming fundamentally different computational mechanisms between bees and humans, and shared mechanisms but substantially different representational richness between adults and infants. By representational richness, we mean that adults simultaneously encode the display at multiple levels, capturing both its holistic appearance and its grid structure, while also forming enhanced, unitized representations of its constituent elements, whereas infants, due to their less developed internal knowledge, appear to encode the display more holistically, without maintaining these alternative interpretations in parallel. More broadly, these results raise the possibility that, while simpler counting‐based learning methods may be highly effective for solving certain learning problems, the capacity to develop implicit sensitivity to conditional contingencies through efficient probabilistic chunking during the construction of rich representations may be critical for humans' remarkable ability to generalize and to perform higher‐order cognition.

## 5 The Statistical Learning of Structure: Contextual Inference and Continual Learning

In order to interact with the world efficiently, the brain develops richly structured internal models of its environment as well as of the body. Examples of such models include our understanding of how shadows and shading come about by the interaction of physical shapes with lighting, allowing us to infer shape from shading (Kersten et al. 2004), abstract semantic phylogenies that allow us to predict properties of animals based on those of related species (Kemp and Tenenbaum 2008), cognitive maps that allow flexible spatial navigation (O'Keefe and Nadel 1978), forward models of motor control that allow us to predict the consequences of our movements (Wolpert et al. 1998), and the repertoire of visual chunks into which we parse visual scenes (Fiser and Aslin 2001; Orbán et al. 2008) (the subject of visual statistical learning studies also described in the previous section).

Statistical learning has been suggested as a key mechanism for learning internal models (Fiser et al. 2010; Kovács and Lengyel 2026). However, the rich structure that we ascribe to these internal models, once they are fully developed, stands in stark contrast with the simplicity with which typical approaches characterize the process of learning itself that gave rise to these internal models in the first place. Even Bayesian theories of statistical learning that have otherwise been successful in explaining why internal models with particular structures emerge as the end result of learning in a range of cognitive domains (Gopnik et al. 2004; Courville et al. 2006; Orbán et al. 2008; Kemp and Tenenbaum 2008; Tenenbaum et al. 2011; Lake et al. 2015) tend to eschew this issue, as they rarely address how these internal models, and in particular their structures, develop over the course of learning.

In general, more often than not, the trial‐to‐trial progression of learning is summarized by the temporal evolution of a single, scalar metric of performance, that is, a “learning curve” (Gallistel et al. 2004). Most of the more sophisticated approaches that go beyond focusing merely on learning‐induced performance changes, and study the cognitive processes underlying these changes, are still too impoverished for capturing changes in the structure of internal models. These approaches typically characterize learning by the temporal evolution of at most a handful of scalar quantities, each simply representing the value associated with a stimulus (Rescorla 1972; Daw et al. 2006) (perhaps also including some measure of uncertainty about these values (Dayan et al. 2000; Daw and Courville 2008)), or the contribution of a particular factor (past stimulus and reward, present stimulus, etc.) to the subject's upcoming choice (Roy et al. 2021). Complex neural network‐based models, incorporating multiple modules (e.g., corresponding to the prefrontal cortex and the basal ganglia) with biologically plausible learning rules updating a large number of synaptic weights have been successfully used to address how task‐relevant context shapes memory and action selection through a combination of various cognitive processes (e.g., working memory and error‐based learning) (O'Reilly and Frank 2006). However, interpreting these models in terms of a structured internal model being learnt can be challenging (Collins and Frank 2013).

More recently, structured statistical descriptions of the learning process have been developed that can capture more sudden leaps in performance and some of its constituent processes (such as attentional lapses and decision biases), but they still do not relate structural changes in behavior to the emergence (and modification) of the structure of internal models (Ashwood et al. 2022; Bruijns et al. 2026). Thus, all these approaches fail to capture the process by which the very structure of internal models arises through experience.

There is one critical facet of structure in our internal models of which the trial‐to‐trial learning has been characterized with some success: context. Our continuous stream of sensorimotor experience is continually segmented into distinct contexts, each represented by a separate memory, or set of memories (Heald et al. 2021; Heald, Lengyel, and Wolpert 2023). Managing this repertoire of context‐dependent memories is critical for our ability to continually learn from experience (in a way that is unparalleled by even today's advanced artificial intelligence algorithms) (Heald, Wolpert, and Lengyel 2023), and is evident in phenomena such as savings, anterograde interference, spontaneous, and evoked recovery, which are ubiquitously observed across multiple cognitive domains in learning paradigms using sufficiently richly varied curricula (Heald, Wolpert, and Lengyel 2023). A particular strand of theories based on the principles of Bayesian nonparametric mixture models (and their temporal extensions, such as hidden Markov models) has successfully explained when and how memories corresponding to different contexts arise and become active (Gershman et al. 2010, 2014; Collins and Frank 2013; Lloyd and Leslie 2013; Heald et al. 2021). The central insight from these theories is that context is inferential: the individual rarely has veridical information about when the context has changed, and if it has changed whether one is experiencing a new context, or a context that has been encountered in the past, and if so, which of those previously encountered contexts is recurring now. Thus, the brain needs to continually infer the current context (“contextual inference” (Heald et al. 2021)), how it is related to past contexts, as well as the statistical contingencies associated with each context. In turn, as perhaps made most explicit in the contextual inference model of motor adaptation (COIN), contextual inference controls when memories get to be created, and which get updated, and expressed in behavior. Importantly, these inferences can sometimes change gradually but sometimes also abruptly, and so changes in performance that may naively be ascribed to “proper” learning (i.e., the creation or updating of memories) may in fact be caused by the dynamics of contextual inference, and thus constitute “apparent” learning (Heald et al. 2021; Heald, Lengyel, and Wolpert 2023).

While contextual inference has successfully accounted for a broad range of behaviorally observable continual learning phenomena, its neural underpinnings remain unknown. Several candidate brain regions and neural phenomena seem relevant (the prefrontal and frontal cortical representations of task sets, or task states, or the remapping of hippocampal place cells (Heald, Wolpert, and Lengyel 2023)), but we do not yet have a detailed mechanistic understanding of how (and which) neural circuits implement contextual inference. Moreover, COIN makes some nontrivial predictions about multiple memories being accessed (for expression or updating) at any one time that, at least seemingly, go against the current canon of the neural basis of learning and memory—according to which only one memory should be updated or expressed (recalled) at any one time (Heald, Wolpert, and Lengyel 2023). Future work will need to better articulate, and ultimately test, these predictions.

## 6 Discussion

We have presented four research avenues examining how the brain uses statistical learning to build structured representations of the sensory world (Figure 1). Two approached the problem from the bottom, characterizing the neuronal substrates of learning‐induced plasticity (Cruces‐Solís et al. 2018; Chen et al. 2023) and the involvement of early stages of the sensory processing hierarchy in probabilistic inference (Tabas et al. 2020; Tabas and Von Kriegstein 2024). The other two approached it from the top, describing the computational operations the brain implements to extract structure (Fiser and Lengyel 2019; Avargues‐Weber et al. 2020) and the inferential operations that organize experience into coherent internal models (Heald et al. 2021). Together, the four lines converge on a unified picture: statistical learning is the brain's primary strategy for transforming a continuous, noisy sensory stream into structured representations of the world.

**FIGURE 1:**
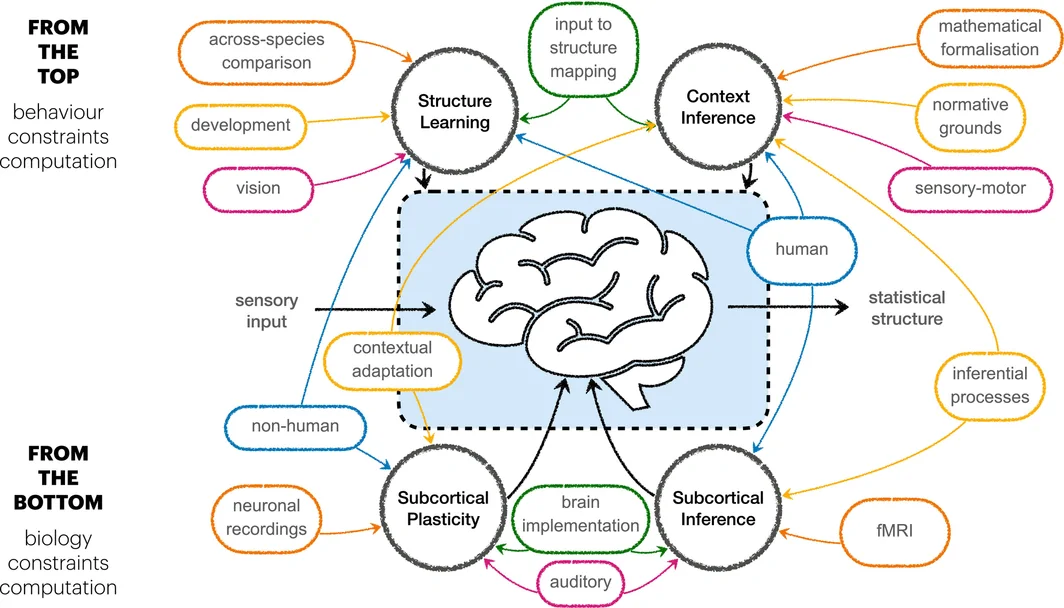
Graphical comparison of the four research lines. The four lines have complementary outlooks on statistical learning, using different sensory modalities (pink boxes), species (blue), and methodologies (orange), and approaching the problem through distinct research aims (yellow).

Considering each of the four research lines in light of the others opens new enquiries and points toward a deeper understanding. For instance, we have described how the frequency tuning of neurons in the auditory midbrain adapts to predictable sound‐context associations (Chen et al. 2023). Although these findings characterize the neural substrates of statistical learning in early sensory structures, they do not address the computational goal of this adaptation. Using the normative lens of the from‐the‐top approaches, one can formally enquire about the principles underlying this plasticity: What are the functional consequences of the observed tuning shifts? Do they reflect optimal inference about the content of the acoustic environment? If so, what generative model of the environment does this computation implicitly assume? Does that model reflect the evolutionary history and ecological niche of the animal?

In the second research line, we have shown that BOLD responses elicited in the human auditory midbrain scale inversely with the explicit prior that participants hold on stimulus occurrence (Tabas et al. 2020, 2024). These results are typically interpreted as hallmarks of Bayesian belief updating (Tabas and von Kriegstein 2021); however, the macroscopic nature of the fMRI data does not provide sufficient physiological detail to draw mechanistic conclusions. Considering these data from the perspective of the first research line, one can begin to constrain mechanistic theories of the underlying neural implementation; for instance, shifts in the frequency tuning of neural populations selective to the stimuli, as reported in (Chen et al. 2023), could give rise to the observed macroscopic responses. Gain modulation offers a further candidate mechanism. These neuronal substrates draw a space of quantitative theories that can be used to design the next generation of experiments to disambiguate between them. Considering the human data from the viewpoint of the from‐the‐top approaches provides further depth: if the brain implicitly extracts sensory structure according to a probabilistic generative model (Fiser and Lengyel 2019, 2022; Heald et al. 2021), how does the inclusion of explicit priors modify that model?

In the third research line, we have described the different probabilistic structures that honeybees, infants, and adults implicitly learn from apparently unstructured visual inputs (Avargues‐Weber et al. 2020). The data revealed intrinsic differences in the statistical learning capabilities across the three groups, but do not explain why such differences exist. The other three research lines provide complementary frameworks with which to address this question at different levels of analysis. For instance, the computational framework of the fourth research line suggests that the differences reflect the structure of the internal generative model that each group possesses (Fiser and Lengyel 2019, 2022): If the generative model does not represent conditional dependencies between elements of the visual landscape, individuals would never be able to identify them, even under optimal inference. The from‐the‐bottom perspectives offer a complementary, physiological angle; for instance, the finding that subcortical plasticity in the auditory midbrain involves corticofugal feedback (Chen et al. 2023) raises the question of whether species and developmental differences may reflect different levels of maturity of the cortico‐subcortical loops. Physiological constraints in the adaptability of the perceptual systems may be the bottleneck that constrains the generative models that the brain can ever acquire.

The last research line described contextual inference, a computational framework that characterizes learning as a continuous process of inferring latent context from sensory experience (Heald et al. 2021). A key insight is that apparent changes in behavior need not reflect genuine learning but may instead arise from shifts in contextual inference alone (Heald et al. 2021; Heald, Lengyel, and Wolpert 2023). This principled framework has successfully accounted for a broad range of continual learning phenomena across cognitive domains, but it remains agnostic about how or where the brain implements its computations (Heald, Lengyel, and Wolpert 2023; Heald, Wolpert, and Lengyel 2023). The second research line provides a methodological baseline that could be used to map these computations to specific brain regions: The fMRI oddball paradigm, which already localizes belief‐updating signals to auditory midbrain and thalamus (Tabas et al. 2020; Tabas and Von Kriegstein 2024), could be adapted to probe which brain structures track context switches and weight competing memories. The first research line may suggest candidate circuit mechanisms through which contextual inference could be implemented: The finding that subcortical plasticity depends on the predictability of associations and involves corticofugal feedback (Chen et al. 2023) points to specific circuit motifs that future work could test. Human neuroimaging and rodent electrophysiological experiments using stimuli drawn from contextual generative models could then shed light on both the neural loci and the synaptic machinery through which the brain implements context‐dependent statistical learning.

These case examples illustrate that bridging across methodological frameworks and levels of analysis has strong potential for advancing our understanding of how the brain implements statistical learning. This potential may itself be a consequence of how statistical learning emerged in the brain in the first place (Cisek and Hayden 2022): If the brain evolved to implement statistical learning under a pressure to behave optimally, then the subsystems implementing it must have co‐evolved to be optimal together, not in isolation. Global optimality of this kind implies that the neuronal mechanisms implementing a computation are shaped by their computational goal, while that goal is itself constrained by the available physiology: a mutual dependence that can only be understood by considering both levels jointly. Viewed in this light, the questions raised by the four research lines are not merely analogous but deeply linked. Why individual neurons in the auditory midbrain shift their frequency tuning in response to predictable associations, why explicit priors modulate macroscopic belief‐updating signals as early as the auditory midbrain, why probabilistic chunking strategies differ across species and development, and how the brain implements contextual inference to organize experience into structured internal models: these may all be facets of a single, globally optimal strategy that the brain has evolved to structure a continuous, noisy sensory stream into discrete, unambiguous representations of the world.

Statistical learning has traditionally been studied through specialized methodological frameworks, each offering a partial view of the phenomenon. To keep advancing in our understanding it is necessary to integrate across the perspectives that study it. Such advancement is important not just to understand how the brain structures the world; it is also important to understand why this process may fail. Deficits in statistical learning may be implicated in conditions such as psychosis (Sterzer et al. 2018) and autism spectrum disorders (Roser et al. 2015), and a mechanistic account that bridges levels of analysis may open more principled targets for diagnosis and intervention. The principles of statistical learning also inform educational strategies for training and skill acquisition (Romberg and Saffran 2013), and offer normative constraints for developing artificial intelligence systems that are efficient, robust, and interpretable (Heald, Wolpert, and Lengyel 2023). Ultimately, understanding how the brain uses statistical learning to structure the sensory world is inseparable from understanding perceptual function and dysfunction.

## Author Contributions

**Alejandro Tabas:** conceptualization, writing – original draft, writing – review and editing. **József Fiser:** writing – original draft, conceptualization, writing – review and editing. **Máté Lengyel:** writing – original draft, writing – review and editing, conceptualization. **Livia de Hoz:** writing – review and editing, conceptualization, writing – original draft.

## Funding

This work was funded by the European Research Council (Grant number 101115798 to A.T.), the Austrian Science Fund (DOI:10.55776/I6793 to J.F.), the Simons Foundation (SFI‐AN‐NC‐SCN‐00007276‐01 to M.L.), the Wellcome Trust (Investigator Award in Science 212262/Z/18/Z to M.L.), the Einstein Foundation (EVF‐2021‐618 to L.d.H.), and the DFG (520617944 and 520284049 to L.d.H.).

## Ethics Statement

The current MS does not involve any original experiment.

## Conflicts of Interest

The authors declare no conflicts of interest.

## Data Availability

The current MS is not associated with any particular dataset.
